# High-resolution optical coherence tomographic imaging of osteoarthritic cartilage during open knee surgery

**DOI:** 10.1186/ar1491

**Published:** 2005-01-17

**Authors:** Xingde Li, Scott Martin, Costas Pitris, Ravi Ghanta, Debra L Stamper, Michelle Harman, James G Fujimoto, Mark E Brezinski

**Affiliations:** 1 Massachusetts Institute of Technology, Department of Electrical Engineering and Computer Science, Research Laboratory of Electronics, Cambridge, MA, USA; 2 Division of Orthopedic Surgery, Brigham and Women's Hospital, Harvard Medical School, Boston, MA, USA; 3 Harvard Medical School, Harvard University, Longwood Avenue, Boston, MA, USA

**Keywords:** birefringence, cartilage imaging, cartilage repair, optical coherence tomography, osteoarthritis

## Abstract

This study demonstrates the first real-time imaging *in vivo *of human cartilage in normal and osteoarthritic knee joints at a resolution of micrometers, using optical coherence tomography (OCT). This recently developed high-resolution imaging technology is analogous to B-mode ultrasound except that it uses infrared light rather than sound. Real-time imaging with 11-μm resolution at four frames per second was performed on six patients using a portable OCT system with a handheld imaging probe during open knee surgery. Tissue registration was achieved by marking sites before imaging, and then histologic processing was performed. Structural changes including cartilage thinning, fissures, and fibrillations were observed at a resolution substantially higher than is achieved with any current clinical imaging technology. The structural features detected with OCT were evident in the corresponding histology. In addition to changes in architectural morphology, changes in the birefringent or the polarization properties of the articular cartilage were observed with OCT, suggesting collagen disorganization, an early indicator of osteoarthritis. Furthermore, this study supports the hypothesis that polarization-sensitive OCT may allow osteoarthritis to be diagnosed before cartilage thinning. This study illustrates that OCT, which can eventually be developed for use in offices or through an arthroscope, has considerable potential for assessing early osteoarthritic cartilage and monitoring therapeutic effects for cartilage repair with resolution in real time on a scale of micrometers.

## Introduction

Osteoarthritis (OA) is the leading cause of chronic disability in developed countries, symptomatically affecting about 14% of the adult population in the United States alone. Among the signs of early OA are collagen disorganization, an increase in water content, a decrease in superficial proteoglycan, and alterations in glycosaminoglycans [1]. The later changes include cartilage loss (thinning effect), fibrillation, and surface erosion. Current imaging technologies are limited in their ability to monitor changes in articular cartilage [2]. Furthermore, symptoms are an unreliable indicator of disease progression [3]. Since the cartilage response to intervention cannot be monitored in a noninvasive or minimally invasive manner, assessing the effectiveness of these drugs and following the progression of the disease remain a challenge. This deficiency is the basis of the current US National Institutes of Health OA initiative to find solutions to this major healthcare dilemma [3]. A diagnostic technique capable of high-resolution imaging of articular cartilage *in vivo *could be invaluable to detect the onset of disease, follow its progression, and monitor therapeutic effectiveness.

Other imaging technologies play an important role in managing OA, but they have limitations. While conventional x-rays have an obvious role in managing arthritis, this technology lacks the resolution to monitor changes within the cartilage [2,4]. Magnetic resonance imaging is invaluable for globally evaluating the joint noninvasively, with a typical clinical resolution of 250–300 μm at 10T [5]. However, the resolution of this technique is problematic, since cartilage is typically less than 2–3 mm thick and the evaluation would rely heavily on the interpretation of a few pixels [6,7]. In addition, its high cost, relatively long imaging time, large size of equipment, and limited availability could limit its widespread clinical use. Arthroscopy is also widely used in the diagnosis of joint disorders [8]. While it provides magnified views of the articular surface, it is unable to assess subsurface.

Optical coherence tomography (OCT) is a recently developed imaging technique that can generate cross-sectional images of tissue microstructure [9,10]. OCT is analogous to ultrasound, but measures the intensity of infrared light rather than sound. It is an attractive imaging alternative for OA because it permits imaging in near-real time with unprecedented high resolution (4–15 μm), 10 to 100 times as fine as that of current clinical imaging modalities. Since OCT is based on fiber-optic systems, the apparatus is compact, roughly the size of an ultrasound unit. Imaging catheters can be constructed with diameters less than 0.006 inches (Lightlab Imaging Inc, Westford, MA, USA; ). Recently, OCT has been adapted for high-resolution imaging in nontransparent tissue. In addition, a variety of spectroscopic techniques can be incorporated, such as absorption, dispersion, and polarization spectroscopy [11-13].

Preliminary work demonstrated the feasibility of OCT in assessing joint cartilage pathologies *in vitro *[11,14]. Microstructures such as fibrillations, cartilage thinning, and new bone growth can be identified on OCT images [14]. Comparison with histology reveals strong correlation between OCT images and corresponding histological sections. In addition, OCT has demonstrated superior qualitative and quantitative performance against both 30- and 40-MHz ultrasound, the current clinical technology with the highest resolution [15,16].

Polarization-sensitivity OCT imaging of articular cartilage has also been performed [11,14]. With this technique, the OCT image changes with change in the polarization state of the incident light. In the previous *in vitro *study, polarization-sensitive changes on OCT images of cartilage were directly correlated with collagen organization [11], as assessed by picrosirius staining. Loss of both polarization sensitivity and collagen organization were noted to take place before cartilage thinning and fibrillation, making it a potential additional marker of early OA in addition to structural imaging. These results have been recently confirmed also in tendons and ligaments, and also in studies with theoretical modeling [17,18]. Through this work, quantitative methods have now been developed and are being studied, including the use of the fast Fourier transform or rate of peak change with rotation of the source optical axis.

This study extends our previous *in vitro *work [11,14]. In this study, observations on the ability of OCT to perform *in vitro *imaging of the human knee were confirmed *in vivo *using a novel handheld probe.

## Materials and methods

**Figure 1:**
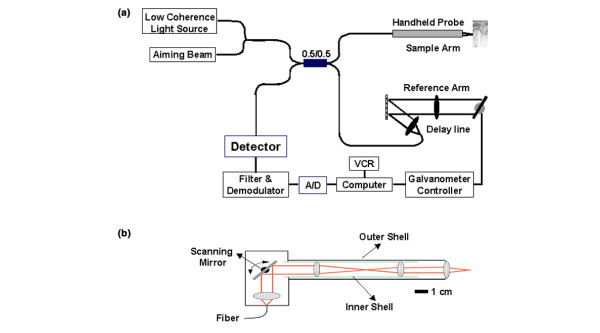
Schematic drawing of the optical coherence tomography (OCT) system and the imaging probe used. The OCT system **(a) **includes a light source with a broad wavelength distribution (called a low-coherence light source), an interferometer (for dividing/recombining the light), and detection electronics. A compact, pen-sized, handheld probe was used for lateral scanning of the articular cartilage, in conjunction with an aiming beam. The handheld OCT imaging probe **(b) **consists of a four-lens relay and a scanning mirror. The outer shell of the probe can be detached for ease of sterilization. A/D, analog-to-digital converter; VCR, video cassette recorder.

The principle behind OCT has been described in detail previously [9,10]. A schematic drawing of the OCT system used in this study is shown in Fig. 1a. In this study, a novel, compact, handheld OCT imaging probe capable of performing lateral scanning of the articular cartilage subsurface during open knee surgeries was used. The probe had dimensions of ~1.5 cm in diameter and ~15 cm in length (see Fig. 1b) and was developed and used to deliver, focus, scan, and detect the returning beam. It consisted of a four-lens relay and a scanning mirror. The measured resolution was approximately 11 μm (axial) and 30 μm (transverse) with a working distance (as defined by the distance between the distal end of the probe and the beam focus) of about 2.5 cm, which provided enough space to perform noncontact imaging. A 532-nm visible beam (green) with a very low power (<0.2 mW) was coupled into the handheld probe for aiming purposes. OCT images were stored in digital format and also recorded on a super VHS tape for future analysis.

The protocol for OCT imaging during open knee surgery was approved by the investigational review board of the Massachusetts Institute of Technology and West Roxbury Veterans Association Hospital. Six patients 65 to 75 years of age who had been diagnosed with severe OA and were scheduled for treatment through partial or total knee replacement surgery were contacted about 4 weeks before surgery and their informed consent was obtained. Patients underwent routine surgical preparation procedures, and OCT imaging did not commence until the articular surface of the femur/tibia was fully exposed. OCT imaging was performed under sterile conditions. Both visually normal and visually abnormal regions were imaged. Imaging planes were marked with sterile dye (methylene blue) for tissue registration. During imaging, the probe did not contact the cartilage surface and the air distance between the probe and the cartilage surface was maintained at ~2.5 cm to insure that the imaged sites remain in focus. Images of 512 × 256 pixels (transverse × axial) were generated at four frames per second. Each OCT image corresponded to a two-dimensional tissue cross section 5 mm wide by 2.6 mm deep. Multiple sites on the articular surface were imaged within the allotted 10-min imaging period. After OCT imaging, surgery resumed as usual. Upon completion of the surgical procedures, the methylene blue dots were re-marked with India ink to improve visualization during post processing. The cartilage was then immediately fixed in 10% buffered formalin and then decalcified with EDTA followed by routine histological processing and stained with Masson trichrome blue.

## Results

**Figure 2:**
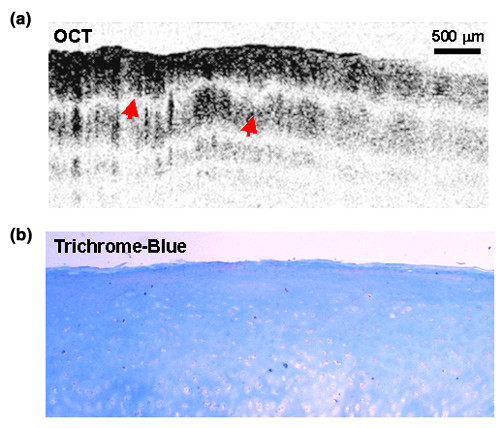
Normal human knee articular cartilage. The optical coherence tomography (OCT) image **(a) **of the cartilage is relatively thick and uniform. The pronounced banding pattern on the OCT image is due to the birefringence of the highly organized structure of the collagen (red arrows). The alternating maximum and minimum intensities are due to changes in back scattering as light travels through the tissue while the plane of light polarization rotates. Previous work has shown that it is due to the presence of organized collagen that alters the polarization state of the light. Note: darker gray scale represents higher-intensity back scattering. The corresponding histology is shown in **(b)**.

A representative OCT image and the corresponding histology of normal knee articular cartilage are shown in Fig. 2. The OCT image (Fig. 2a) reveals that the cartilage was thick and uniform with a rather smooth surface. The same characteristics can also be seen in the histology as shown in Fig. 2b. A banding pattern is seen in the OCT image (Fig. 2a, red arrows). Previous work showed that this pattern represents alternating maximum and minimum intensities of back scattering, which results from rotation of the polarization state of back-reflected light as it passes through the organized collagen. During the imaging process, it was noted that the position of the bands moved as the polarization state of the incident light was changed (induced by moving the fiber in the sampling arm).

**Figure 3:**
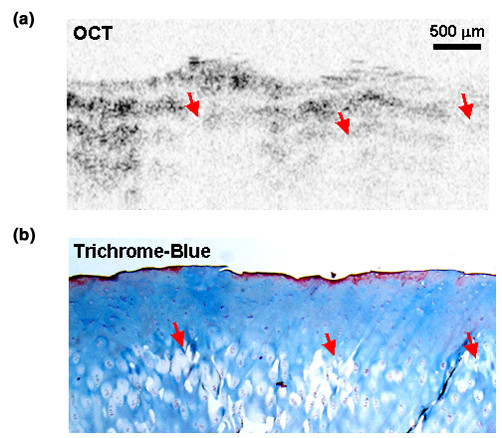
Representative optical coherence tomography (OCT) image **(a) **and the corresponding histology **(b) **of mild to moderate osteoarthritic knee cartilage. Regions of lost back scattering are noted in the OCT image. These regions correlate with abnormalities detected on the corresponding histology (b). Areas of hypocellularity are indicated by the red arrows.

Fig. 3 illustrates a representative OCT image (Fig. 3a) and the corresponding histology (Fig. 3b) of moderately diseased cartilage. Regions of diminished back scattering are noted in the OCT image, which correlate with areas of hypocellularity and diminished matrix in histological preparations. On the OCT image, the banding pattern is disrupted and correlates with histologically abnormal staining and cellularity.

**Figure 4:**
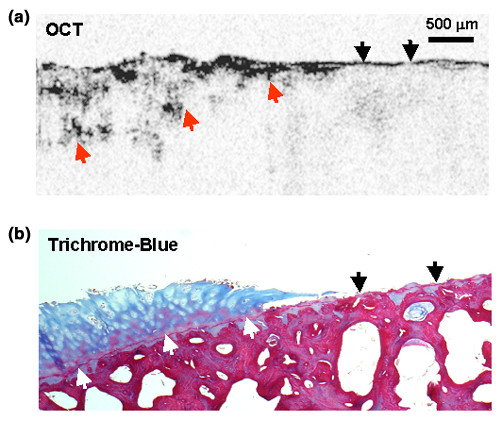
An optical coherence tomography (OCT) image **(a) **and the corresponding histology **(b) **of severely degenerated cartilage. The heterogeneity of the cartilage and loss of the polarization sensitivity are noted. The subchondral bone interface is indicated by either white (a) or red (b) arrows. Black arrows indicate areas in which cartilage is absent with the bone exposed.

Fig. 4 shows an OCT image (Fig. 4a) and the corresponding histology (Fig. 4b) of severely diseased cartilage. Distinctive thinning of the cartilage was observed only on the left portion of both OCT image and histology. In addition, an irregular cartilage surface is seen in the OCT image, with multiple fibrillations evident in the corresponding histology. The OCT image is highly heterogeneous and the cartilage and bone interface are poorly identified. No banding appearance or polarization sensitivity was observed on this image. On the right portion of the OCT image and the histology section, cartilage is absent and the bone is exposed to the surface.

**Figure 5:**
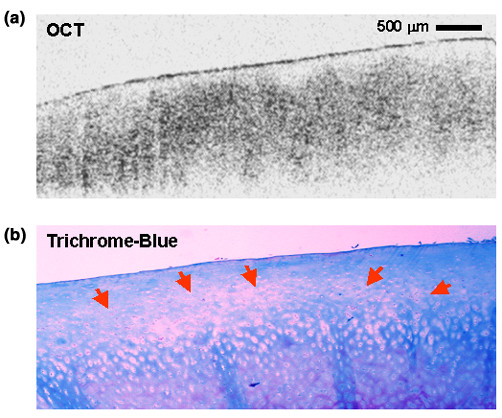
Optical coherence tomography (OCT) image **(a) **of cartilage with evidence of early degenerative changes and the corresponding histology **(b)**. Areas of hypocellularity are indicated with red arrows.

An OCT image of thick cartilage with no evidence of surface erosion and early degenerative changes is shown in Fig. 5. The OCT structural image is relatively homogeneous but the banding pattern is lost. The abnormal region seen on histology consists of an area of hypocellularity over a region of hypercellularity.

**Figure 6:**
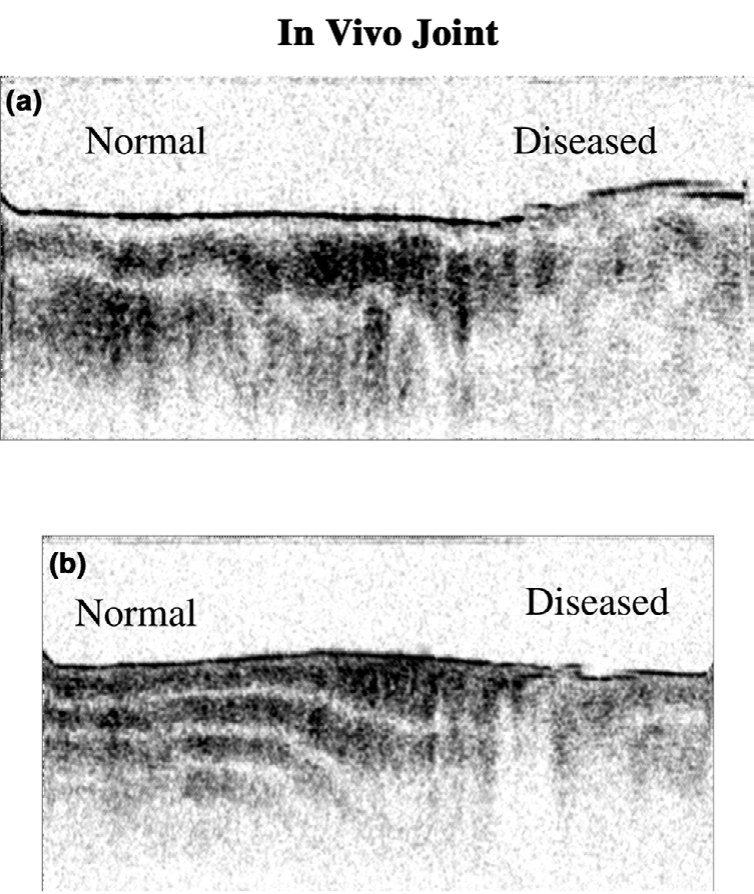
Optical coherence tomography image of cartilage from femur and patella consisting of adjacent areas of normal and diseased tissue. The banding pattern is attenuated and lost in diseased areas (on the right portion of each image). In addition, back-scattering intensity is abruptly reduced.

Fig. 6 shows normal and diseased cartilage in close approximation in two sections of cartilage. The region on the left of both images is presumed normal cartilage, while on the right, the polarization sensitivity and back-scattering intensity abruptly changes. In addition, since these two samples come from the femur (Fig. 6a) and patella (Fig. 6b), respectively, the figure confirms that the polarization phenomenon exists in areas other than the tibia.

## Discussion

The current study demonstrates that osteoarthritic structural changes in cartilage can be visualized with OCT *in vivo *using a handheld probe. Structural changes including cartilage thinning and fibrillations were observed at a resolution substantially higher than that of any current clinical imaging technology. While normal cartilage demonstrates a banding pattern with a relatively homogeneous intensity (as seen in Fig. 2), areas of hypocellularity appear to lose this banding pattern (as seen in Fig. 3). These changes are dramatic enough to distinguish between adjacent areas of healthy and diseased tissue (as in Fig. 6). These results indicate that OCT may be able to be used by surgeons to aid in the evaluation of the microstructural integrity of articular cartilage during surgical procedures.

It can ultimately be envisioned that OCT imaging will be performed with a surgical arthroscope or a needle arthroscope for assessing the articular cartilage in a minimally invasive fashion. Future efforts will be on the development of a small OCT arthroscope capable of being either used in combination with or integrated into a standard arthroscope. Endoscopic imaging using an OCT probe introduced through the accessory port of an endoscope has been demonstrated in the human gastrointestinal tract [19,20].

The collagen matrix in healthy cartilage is a highly organized structure [21,22]. The banding pattern seen on the OCT images (e.g. Figs 2, 3, and 6) are due to tissue birefringence and are related to collagen organization [11,14]. Changes in collagen organization, although not necessarily in collagen content, are among the earliest changes in OA [1]. It has been shown in animals that a decrease in birefringence, evident on histological evaluation, precedes fibrillations and can even be noted after chronic long-distance running [23,24]. The diminishing and absent banding pattern on the OCT images (e.g. Figs 3,4,5,6), an observation supported by *in vitro *work, represents a reduction and loss of the birefringence of the cartilage, which is caused by the reduction or loss of collagen structural organization [14]. This has recently been confirmed in experimental models of OA in the rat [25,26]. That study indicated that changes in the birefringent properties of cartilage (as with OA) are reflected in the polarization sensitivity of OCT images. In the current study, polarization changes were not quantitatively measured. However, as the fiber of the sample arm moved, it would induce a polarization state shift, allowing quick assessment of the polarization sensitivity of the area being imaged. Protocols are now available using fast Fourier transforms to quantitate single-detector OCT.

## Conclusion

A true clinical need exists for monitoring therapeutic intervention with regard to osteoarthritic cartilage. This study demonstrates real-time, high-resolution OCT imaging of articular tissues *in vivo *during joint replacement surgery at resolutions on a scale of micrometers. Abnormalities such as cartilage thinning and fibrillations were detected and qualitatively correlated with the corresponding histology. In addition, birefringence changes between osteoarthritic and normal cartilage were noted in this study, indicative of a loss of collagen organization. OCT represents a promising new technology for the evaluation of articular cartilage *in vivo*.

## Abbreviations

OA = osteoarthritis; OCT = optical coherence tomography.

## Competing interests

The author(s) declare that they have no competing interests.

## Authors' contributions

XL designed and constructed the OCT system. SM performed studies in patients, which included gaining their consent and postoperative observation. CP assisted in the construction of the OCT system. RG assisted with the construction of the handheld probe. DS advised on histological preparations. MH processed the tissues. JF consulted on the design of the OCT system. MB was involved with the engineering design, OCT protocol, evaluation of data, and writing of manuscript. All authors read and approved the final manuscript.
